# What Are the Infection Prevention Behaviors of Kidney Transplant Recipients and the Factors Related to These?

**DOI:** 10.1111/jocn.17522

**Published:** 2024-11-05

**Authors:** Yaprak Sarıgöl Ordin, Bahar Karakılçık

**Affiliations:** ^1^ Surgical Nursing Department, Faculty of Nursing Dokuz Eylul University Izmir Turkiye; ^2^ Surgical Nursing Department, Health Sciences Institute Dokuz Eylul University Izmir Turkiye

**Keywords:** hygiene, infection prevention behaviours, kidney transplant, self‐control, self‐management

## Abstract

**Background:**

The infection prevention behaviours of kidney transplant recipients have been investigated, but the factors affecting these have not. Therefore, the present study aimed to examine the infection prevention behaviours of kidney transplant recipients and the factors related to these.

**Methods:**

The sample of this descriptive, cross‐sectional study included 148 kidney transplant recipients being followed in a kidney transplant outpatient clinic. Data were collected with a sociodemographic and clinical characteristics form, the Adherence to Measures against Infections Questionnaire and the Self‐Control and Self‐Management Scale between November 2022 and May 2023.

**Results:**

The mean age of the patients was 50.29 ± 13.11 years. The rate of self‐reported hand hygiene was 51%. Age caused a significant difference in receiving a vaccine against pneumonia and wearing a mask. The rate of self‐reported behaviour of rinsing vegetables was higher among the married patients compared to single patients. Self‐management created a significant difference in receiving a vaccine against influenza and pneumonia, eating salad in a café or restaurant and wearing a mask outside home and consuming raw food, delicatessen products and street food (*p* < 0.05).

**Conclusion:**

This study revealed that the kidney transplant recipients displayed incomplete infection prevention behaviours including hand hygiene. Infection prevention behaviours differed in terms of age and self‐control and self‐management. Education and interventions are needed to improve the infection prevention behaviours of kidney transplant recipients.

**Relevance to Clinical Practice:**

This study illustrates the characteristics of, and relationship between, sociodemographic factors and self‐management of infection prevention behaviours in kidney transplant recipients.

**Reporting Method:**

Strengthening the reporting of cross‐sectional studies (STROBE) was followed.

**Patient Contribution:**

The study demonstrates that there is a need for interventions that will improve both the self‐management levels and infection protection behaviours of patients. Studies conducted in this direction will lead to a decrease in the infection rates of patients.

**Summary**

- Why is this study needed?
  ○ It is crucial to determine whether organ transplant recipients display infection prevention behaviours. Prior studies have examined certain infection prevention behaviours, but have not primarily focused on the factors affecting these.
  ○ There have not been any studies examining the relation between self‐management and infection prevention behaviours.
  ○ Knowing these factors will help determine risk groups in terms of adherence to infection prevention behaviours.
- What are key findings?
  ○ The hand hygiene behaviours displayed by the kidney transplants recipients were not adequate in this study.
  ○ As the patients' age increased, so did the rate of receiving a pneumonia vaccine.
  ○ A higher rate of married patients reported rinsing vegetables compared to single patients.
  ○ Good self‐management was found to lead to a difference in infection prevention behaviours.
- How should the findings be used to influence policy/practice/research/education?
  ○ Programmes and interventions are needed to improve the infection prevention behaviours of kidney transplant recipients.
  ○ In particular, it appears that improving the self‐management of kidney transplant recipients will improve infection prevention behaviours.

## 1 Introduction

Infections appear both early after organ transplantation and in the long‐term and negatively affect patient outcomes (Koçak 2016). Infections in kidney transplant recipients lead to graft dysfunction and rejection, systemic diseases, risk of cancer and increased mortality (Dinkçi et al. 2022; Mella et al. 2022). The leading cause of death after kidney transplantation is cardiovascular complications, followed by infections (Awan et al. 2018). Infections are responsible for 55% of readmissions to hospital after kidney transplantation (Boubaker et al. 2011).

Infections are a complication that can be prevented or reduced substantially. Immunosuppression, invasive intervention, type of donor and epidemiological exposures increase the risk of infections (Tarhini et al. 2023). Direct contact of patients with individuals carrying potential pathogens, contaminated food, air pollution, water pollution, soil and hospital environments are the sources of infections (Fishman 2019). Kidney transplant recipients should be provided with counselling to minimise the risk of infections. Guidelines recommend preventing direct contact‐related infections and respiratory infections, achieving hand hygiene, avoiding tobacco use and ensuring water safety, food safety, safe contact with animals, safe sexual life, safe travel, work and school safety and safe contact with infected individuals for the minimisation of infection risk (Avery and Michaels 2019; Blair 2019). According to research, 97% of kidney transplant recipients are careful with hand hygiene (Dolgun et al. 2017), 78% have partial adherence to measures against infections, 16% avoid contact with infected individuals, 8% do not use mass transportation and 5% avoid crowded places (Hedayati, Shahgholian, and Ghadami 2017). It is stated in the literature that 18% of the kidney transplant recipients follow all food safety recommendations (Lindup et al. 2020). It has also been reported that women have a higher level adherence to measures against infections, but that as time from transplantation increases, adherence decreases (Hedayati, Shahgholian, and Ghadami 2017).

As organ transplant recipients face many complications, especially infections and rejection after transplantation, they are regularly followed up. Guidelines for protection from infections in organ transplant patients focus on the behaviours that patients need to pay most attention to (Avery and Michaels 2019; Blair 2019). In line with these guidelines, organ transplant teams provide training to patients after organ transplants. How the patients adapt to these behaviours has an effect on how well they are protected from, and the development of, infections. In addition, whether and to what extent patients are able to adapt to these behaviours is a variable factor for the prevention of infections. Organ transplant recipients are able to develop health promotion behaviours and take responsibility for protecting their own health during the transplantation process (Lin et al. 2011; Turner, Burns, and Tranter 2018; Weng et al. 2010). Individuals who have achieved self‐management and adjusted to changes in their lives can play an active role in looking after their transplanted kidney (Kobus et al. 2011). Prior studies have examined specific infection prevention behaviours, but have not primarily focused on factors affecting these. Knowing these factors will help determine risk groups in terms of adherence to infection prevention behaviours. The results of the present study will provide a basis for interventional studies to improve the adherence of risk groups to infection prevention behaviours. There have not been any studies examining the relation between self‐management and infection prevention behaviours. The aim of the present study was to examine the infection prevention behaviours of kidney transplant recipients and factors relating to these (age, time after transplantation, gender and marital status). The secondary aim of the study was to examine the relation between infection prevention behaviours and self‐management in kidney transplant recipients.

## 2 Methods

### 2.1 Study Design

The study had a descriptive and cross‐sectional design. The infection prevention behaviours and self‐control and self‐management levels of the kidney transplant recipients were evaluated by conducting a single face‐to‐face interview with each recipient. Strengthening the reporting of cross‐sectional studies (STROBE; Appendix S1) was followed (von Elm et al. 2007).

### 2.2 Study Sample

The study was conducted in a kidney transplant outpatient clinic of a university hospital in the western part of Türkiye. Data were collected between November 2022 and May 2023. There were 440 kidney transplant patients in the outpatient clinic where the study was performed. The study sample included 148 kidney transplant patients. The *inclusion criteria* were being 18 years old or more, speaking and writing Turkish, being able to communicate and agreeing to participate in the study. The *exclusion criteria* were being illiterate, having the diagnosis of a psychiatric disease, having multiple organ transplants, being a hospital inpatient and having an active infection.

The study power was found to be 92% based on the effect size of 0.50 in the analyses of the difference between having and not having a pneumonia vaccine and in terms of mean age by using the G*Power program.

The centre where the present study was performed offers education about infection prevention behaviours at discharge. This education can be repeated at follow‐ups when patients require this. The education includes hand hygiene, wearing a mask in crowded places, water safety, food safety, safety of pets, having a safe sexual life, safety when travelling, the residential area and contact with infected individuals.

### 2.3 Data Collection Tools

Data about clinical features of the kidney transplant recipients were obtained from the patient records kept in the kidney transplant outpatient clinic. Additionally, data were gathered by the researcher by using a sociodemographic and clinical characteristics form, the Adherence to Measures against Infections Questionnaire and the Self‐Control and Self‐Management Scale (SCMS) during face‐to‐face interviews. Data collection was performed between November 2022 and May 2023 when the patients presented to the kidney transplant outpatient clinic for their routine check‐ups. The patients were taken to a quiet room next to the outpatient clinic after their check‐ups had been completed. The patients were provided with information about the study and their written informed consent was obtained. They were then given the self‐report data collection tools and asked to respond to the items in the tools during a face‐to‐face interview. The patients could ask the researcher questions about any items they did not understand or were unsure about.

### 2.4 Sociodemographic and Clinical Characteristics Form

The sociodemographic and clinical characteristics form was prepared by the researchers in light of the literature and was composed of 21 questions. Of these questions, 11 were about sociodemographic characteristics, including age, gender, education, marital status, employment status, occupation, income levels, family types, number of children, place of residence and people staying with the kidney transplant recipients. The remaining 10 questions were about clinical features including the aetiology of the kidney transplantation, date of transplantation, renal replacement therapy, number of transplants received, type of donors, degree of relation with donors, receiving immunotherapy, immunosuppressants used, other medications used and chronic diseases.

### 2.5 Adherence to Measures Against Infections Questionnaire

The Adherence to Measures against Infections Questionnaire was created by the researchers in light of the literature and by taking account of the relevant guidelines in order to determine the adherence of the kidney transplant recipients to measures against infections (Avery and Michaels 2019; Baker et al. 2017; Blair 2019; Kasiske et al. 2010; Voora and Adey 2019; Whittaker et al. 2012). The questionnaire assesses patients' infection prevention behaviours based on their self‐report. It is composed of 33 questions about vaccination status, hand hygiene, water safety, food safety, safe contact with pets, safety of pets, a safe sexual life, safety when travelling, piercings, residential area and contact with infected individuals. To achieve content validity for the questionnaire, expert opinion about the content was obtained from five specialists (three transplant nurses, one nephrologist and one infection specialist). The content validity index (CVI) and content validity ratio (CVR) were calculated using Lawshe and Davis's technique. The CVI and CVR of the questionnaire were found to be 1.0 after expert opinion was obtained. The questionnaire was piloted with five patients and its latest version was created in accordance with the feedback received.

### 2.6 Self‐Control and Self‐Management Scale (SCMS)

The SCMS was developed by (Mezo 2009) to measure the self‐control and self‐management of individuals (Mezo 2009). The scale comprises 16 items and three subscales: self‐monitoring, self‐evaluation and self‐reinforcing. It is a six‐point scale with 0 corresponding to ‘doesn't describe me at all’ and five corresponding to ‘describes me completely’. Items 7, 8, 9, 10 and 11 are reverse‐scored in the reverse order. The total score for the scale ranges from 0 to 80; as the total score increases, so do self‐control and self‐management levels (Mezo 2009).

The validity and reliability of the SCMS were tested on university students. The Cronbach's alpha value was reported to be 0.87 for the scale, 0.80 for the self‐monitoring subscale, 0.73 for the self‐evaluation subscale and 0.81 for the self‐reinforcing subscale (Ercoşkun 2016). In the present study, the Cronbach's alpha value for the scale was found to be 0.77 and the Cronbach's alpha values for the self‐monitoring, self‐evaluation and self‐reinforcing subscales were found to be 0.69, 0.80 and 0.65, respectively.

### 2.7 Data Analysis

Data were analysed with the Statistical Package for the Social Sciences (SPSS) 29.0. Descriptive statistics (numbers, percentages, mean values, etc.) were used to determine the sociodemographic characteristics of the patients. The Kolmogorov–Smirnov and Shapiro–Wilk tests were utilised to determine whether the data were normally distributed. For evenly distributed data about the variables, the independent groups *t*‐test was utilised to identify the differences between the groups. In the absence of an even distribution, the Mann–Whitney *U*‐test and Kruskal–Wallis test were adopted to determine differences. The chi‐squared test was used for the analysis of categorical variables. Logistic regression analysis was performed on the variables which were found to have a difference in the analyses performed. In the ‘rinsing vegetables’ dependent variable, no regression analysis was performed as the number of individuals saying ‘yes’ in the single group was 0 in the distribution according to marital status.

### 2.8 Ethical Considerations

Ethical approval was obtained from the Ethics Board for Non‐Interventional Research (approval date: 12 October, 2022; approval number: 2022/32‐14) and permission was obtained from the hospital administration where the study was carried out. After the participants were provided with information about the study, their written informed consent was obtained.

## 3 Results

The mean age of the kidney transplant recipients was 50.29 ± 13.11 years (minimum = 20.85 years; maximum = 85 years) and the mean time after transplantation was 12.77 ± 6.41 years (minimum 0.62 years; maximum 28.40 years) (Table 1). The mean number of the medications taken by the patients was 5.82 ± 1.48 (minimum–maximum = 3–10). The immunosuppressive therapy taken by the highest percentage of the patients included corticosteroids, tacrolimus (twice a day) and mycophenolate mofetil at the rate of 36.48% (*n* = 54). The patients' total self‐control and self‐management scale score was found to be 4.36 ± 0.47 (minimum 2.94, maximum 5.00). The mean self‐monitoring subscale score was 4.32 ± 0.64 (minimum 2.33, maximum 5.00), the mean self‐evaluation subscale score was 4.67 ± 0.62 (minimum 2.00, maximum 5.00) and the mean self‐reinforcement subscale score was 4.12 ± 0.71 (minimum 2.20, maximum 5.00).

**TABLE 1:** Sociodemographic and clinical features (*n* = 148).

Features	Mean ± SD
Age (min–max)	50.29 ± 13.11 (20.85–85.00)
Time after transplantation (years)	12.77 ± 6.41 (0.62–28.40)
Number of the medications	5.82 ± 1.48 (3–10)

	*n* (%)
Gender	
Female	60 (40.54%)
Male	88 (59.46%)
Education	
Primary education	40 (27.04%)
High school	52 (35.13%)
University or a higher education level	56 (37.83%)
Marital status	
Married	113 (76.35%)
Single	35 (23.5%)
Employment status	
Full‐time	44 (29.74%)
Part‐time	4 (2.70%)
Unemployed	100 (67.56%)
Aetiology of kidney transplantation	
Hypertension	48 (32.43%)
Diabetes mellitus	14 (9.45%)
Chronic glomerulonephritis	12 (8.10%)
Polycystic CRD	13 (8.78%)
FSG	9 (8.08%)
FAF + amyloidosis	5 (3.37%)
Unknown	25 (16.89%)
Other	22 (12.9%)
The number of kidney transplantations	
1	140 (94.59%)
2	7 (4.72%)
3	1 (0.69%)
Type of donor	
Living	87 (58.78%)
Cadaver	61 (41.22%)
Relation with the living donor (*n* = 87)	
Spouse	20 (22.99%)
Mother	34 (39.08%)
Father	11 (12.64%)
Sibling	20 (22.99%)
Aunt	1 (1.15%)
Uncle	1 (1.15%)
Presence of a chronic disease	
Yes	124 (83.78%)
No	24 (16.22%)
Chronic diseases (*n* = 124)	
Diabetes mellitus	21 (16.94%)
Hypertension	71 (57.26%)
Coronary heart disease	7 (5.65%)
Diabetes mellitus + hypertension	3 (2.41%)
Other	22 (17.74%)

*Note:* Other: 5 vesicourethral reflux, 4 immunoglobulin A nephropathyi, 4 nephrotic syndrome, 3 renal cell carcinoma, 3 nephrolithiasis, 1 neurogenic bladder and 1 solitary kidney, 1 nephrotic syndrome, 1 SLE, 1 IMMUNE complex glomerulonephritis (membranoproliferative glomerulonephritis).

Abbreviations: CRD, chronic renal disease; FMF, familial Mediterranean fever; FSG, focal segmental glomerulosclerosis; SLE, systemic lupus erythematosus; VUR, vesicourethral reflux.

The results regarding infection prevention behaviours are based on self‐reported data. The rate of self‐reported hand hygiene (frequent hand washing) was 51.35% (*n* = 79). In addition, the top three most self‐reported hand hygiene behaviours were washing hands after using the toilet (50%; *n* = 74), washing hands before and after meals (42.56%; *n* = 63) and washing hands upon arriving at home (32.43%; *n* = 48). The mean duration of washing hands was 13.99 ± 7.66 s (min–max = 10–30 s). Only 22.98% of the kidney transplant recipients (*n* = 34) had a pet; these included birds (*n* = 14), dogs (*n* = 12) and cats (*n* = 8). Of the recipients with a pet, 50% (*n* = 17) took their pets to a veterinarian for routine examinations and 41.17% (*n* = 14) had their pets vaccinated. While 42.56% of the recipients (*n* = 63) travelled once a year, 57.44% (*n* = 85) reported that they did not travel much. During their travels, 39.87% (*n* = 59) took preventive measures, whereas 60.13% (*n* = 89) did not take any precautions. In addition, 48.64% (*n* = 72) had received information about vaccination. The vaccination status of the kidney transplant recipients is presented in Table 2 and the hygiene behaviours of the recipients are presented in Table 3.

**TABLE 2:** Immunisation status of kidney transplant recipients (*n* = 148).

Flu vaccine status (*n* = 148)		Frequency of flu vaccine (*n* = 90)	
Yes	90 (61%)	Every year	76 (84%)
No	58 (39%)	2–3 years	14 (16%)

Pneumococcal vaccine status (*n* = 148)		Frequency of pneumococcal vaccine (*n* = 72)	
Yes	72 (49%)	5 years	57 (79%)
No	76 (51%)	10 years	15 (21%)

Travel to endemic places (*n* = 148)		Immunisation receiving before travel (*n* = 6)	
Yes	6 (4%)	Yes	0 (0%)
No	142 (96%)	No	6 (100%)

**TABLE 3:** Hygiene behaviours of kidney transplant recipients (*n* = 148).

Nutrition hygiene	*n* (%)	Personal hygiene	*n* (%)
Drinking water		Having a separate room	
Tap water	15 (10.13%)	Yes	120 (81.1%)
Packaged water	67 (45.27%)	No	28 (18.9%)
Filtered water	66 (44.60%)		
Wearing gloves when in contact with soil		Having a separate toilet and bathroom	
Yes	29 (19.59%)	Yes	36 (24.3%)
No	119 (80.41%)	No	112 (75.7%)
Rinsing vegetables and fruit		Being careful when a family member has a viral infection at home	
Yes	135 (91.21%)	Yes	130 (87.8%)
No	13 (8.79%)	No	18 (12.2%)
Wearing gloves when in contact with uncooked meat and preparing meat products		Wearing a mask at home	
Yes	10 (6.76%)	Yes	7 (4.7%)
No	138 (93.24%)	No	141 (95.3%)
Consuming raw food		Wearing a mask outside home	
Yes	48 (32.44%)	Yes	40 (27%)
No	100 (67.56%)	No	108 (73%)
Consuming canned food		Being in crowded places	
Yes	33 (22.29%)	Always	18 (12.16%)
No	115 (77.71%)	Frequently	21 (14.19%)
		Sometimes	61 (41.21%)
		Rarely	17 (11.49%)
		Never	31 (20.95%)
Washing food cans		Awareness about how to protect against sexually transmitted diseases	
Yes	9 (6.08%)	Yes	136 (91.9%)
No	139 (93.92%)	No	12 (8.1%)
Place of buying milk and milk products		Having manicure	
Market	91 (61.48%)	Yes	0 (0%)
Creamery	24 (16.23%)	No	148 (100%)
Farmers I know	33 (22.29%)		
Consuming unpasteurised milk and milk products		Piercing	
Yes	8 (5.40%)	Yes	1 (0.7%)
No	140 (94.60%)	No	147 (99.3%)
Consuming delicatessen products		Having a tattoo	
Yes	29 (19.60%)	Yes	2 (1.4%)
No	119 (80.40%)	No	146 (98.6%)
Eating salad in a café or restaurant		Place of swimming preferred	
Yes	90 (60.81%)	Pool	4 (2.7%)
No	58 (39.19%)	Sea	77 (52%)
		Thermal pool	1 (0.7%)
		I don't swim	66 (44.6%)
Consuming street food			
Yes	80 (54.05%)		
No	68 (45.95%)		

Table 4 shows the results of the analyses performed according to the patients' age, time after transplantation, gender, marital status and total self‐control/self‐management and subscale scores. Logistic regression analysis was performed on the variables which were found to have a difference. According to the logistic regression analysis, the mean self‐evaluation and self‐reinforcing scores were, respectively, 0.50 and 0.54 times greater in the patients who had had the influenza vaccine than in the patients who had not had the influenza vaccine (4.78 **±** 0.48 vs. 4.49 ± 0.76, 95% CI, 0.272–0.936, *p* = 0.030; 4.21 ± 0.72 vs. 3.97 ± 0.68, 95% CI, 0.308–0.961, *p* = 0.036, respectively). The mean age and mean self‐reinforcing score were, respectively, 0.57 and 0.56 times greater in the patients who had had the pneumonia vaccine than in the patients who had not had the pneumonia vaccine (53.03 **±** 12.71 vs. 47.71 **±** 13.03, 95% CI, 0.939–0.996, *p* = 0.027; 4.25 ± 0.73 vs. 3.99 ± 0.67, 95% CI, 0.320–0.987, *p* = 0.045, respectively). The mean self‐monitoring score was 1.99 times greater in the patients who avoided raw food than in the patients who ate raw food (4.41 ± 0.59 vs. 4.11 **±** 0.68, 95% CI, 1.049–3.796, *p* = 0.035). The self‐reinforcing score was 2.18 times greater in the patients who did not eat salad in cafés or restaurants than in the patients who did eat salad in cafés or restaurants (4.33 ± 0.67 vs. 3.98 ± 0.71, 95% CI, 1.199–3.951, *p* = 0.011). The self‐reinforcing score was 1.77 times greater in the patients who avoided street food than in the patients who ate street food (4.28 ± 0.70 vs. 3.98 ± 0.69, 95% CI, 1.005–3.094, *p* = 0.011). The mean age was 0.95 times greater in patients who wore a mask outside their homes than in the patients who did not wear a mask outside their homes (56.10 **±** 11.45 vs. 48.15 **±** 13.07, 95% CI, 0.914–0.980, *p* = 0.002, Table 5).

**TABLE 4:** Differences in infection prevention behaviours in terms of age, gender, marital status, time after kidney transplantation and self‐control/self‐management levels.

	Receiving influenza vaccine	Receiving pneumonia vaccine	Consumed drinking water	Wearing gloves when in contact with soil	Rinsing vegetables	Uncooked meat	Raw food	Canned food	Milk and milk products	Pasteurised milk	Delicatessen products	Eating salad in a café or restaurant	Consuming street food	Having/Not having a pet	Wearing a mask outside home
Age	*t* = 1.398 *p* = 0.164	***t*** = **2.511** ***p*** = **0.013** *	KW = 2.84 *p* = 0.319	*z* = 1474.500 *p* = 0.225	*z* = 767.500 *p* = 0.456	*z* = 614.000 *p* = 0.708	*t* = −1.771 *p* = 0.079	*t* = −1.204 *p* = 0.230	KW = 2.766 *p* = 0.429	*z* = 603.500 *p* = 0.712	*z* = 1880.000 *p* = 0.455	*t* = −0.690 *p* = 0.093	*t* = −1.772 *p* = 0.078	*t* = −0.463 *p* = 0.644	*t* = **3.393** ***p*** = **0.001** *
Time after transplantation	*z* = 2848.500 *p* = 0.349	*z* = 2598.000 *p* = 0.597	KW = 4.355 *p* = 0.113	*z* = 1737.500 *p* = 0.954	*z* = 1091.000 *p* = 0.148	*z* = 563.00 *p* = 0.332	*z* = 2308.000 *p* = 0.706	*z* = 2112.500 *p* = 0.322	Kw = 6.046 *p* = 0.109	*z* = 620.000 *p* = 0.611	*z* = 1961.500 *p* = 0.254	*z* = 2583.000 *p* = 0.916	*z* = 2577.000 *p* = 0.582	*z* = 1847.000 *p* = 0.678	*z* = 2530.000 *p* = 0.110
Gender	*X* ^2^ = 1.049 *p* = 0.306	*X* ^2^ = 3.632 *p* = 0.057	*X* ^2=^0.183 *p* = 0.913	*X* ^2^ = 2.493 *p* = 0.114	*X* ^2=^0.018 *p* = 0.892	*X* ^2^ = 1.074 *p* = 0.300	*X* ^2=^2.005 *p* = 0.157	*X* ^2^ = 1.340 *p* = 0.247	*X* ^2^ = 2.897 *p* = 0.408	*X* ^2^ = 0.866 *p* = 0.270	*X* ^2^ = 1.887 *p* = 0.170	*X* ^2^ = 0.114 *p* = 0.735	*X* ^2^ = 0.971 *p* = 0.325	*X* ^2^ = 0.081 *p* = 0.766	*X* ^2^ = 2.608 *p* = 0.106
Marital status	*X* ^2^ = 0.232 *p* = 0.630	*X* ^2^ = 0.349 *p* = 0.555	*X* ^2^ = 1.042 *p* = 0.594	*X* ^2^ = 1.321 *p* = 0.250	***X*** ^**2**^ = **3.095** ***p*** = **0.039** *	*X* ^2^ = 0.011 *p* = 0.701	*X* ^2^ = 0.225 *p* = 0.635	*X* ^2^ = 0.001 *p* = 1.000	*X* ^2^ = 2.981 *p* = 0.395	*X* ^2^ = 0.008 *p* = 1.000	*X* ^2^ = 0.098 *p* = 0.754	*X* ^2^ = 0.771 *p* = 0.380	*X* ^2^ = 1.004 *p* = 0.316	*X* ^2^ = 1.265 *p* = 0.243	*X* ^2^ = 0.728 *p* = 0.393
Self‐control/self‐management	***z*** = **2066.500** ***p*** = **0.032** *	***z*** = **2049.500** ***p*** = **0.008** *	KW = 4.146 *p* = 0.126	*z* = 1790.000 *p* = 0.755	*z* = 870.500 *p* = 0.962	*z* = 797.000 *p* = 0.423	*z* = 2840.000 *p* = 0.071	*z* = 2183.00 *p* = 0.187	KW = 3.135 *p* = 0.371	*z* = 667.500 *p* = 0.361	*z* = 2085.500 *p* = 0.081	***z*** = **3290.500** ***p*** = **0.007** *	*z* = 3429.000 *p* = 0.006*	*z* = 1772.000 *p* = 0.448	***z*** = **1640.500** ***p*** = **0.025** *
Self‐monitoring	*z* = 2556.000 *p* = 0.830	*z* = 2453.500 *p* = 0.272	KW = 3.300 *p* = 0.192	*z* = 1709.500 *p* = 0.938	*z* = 698.500 *p* = 0.219	*z* = 924.000 *p* = 0.070	***z*** = **3008.000** ***p*** = **0.012** *	*z* = 2152.000 *p* = 0.235	KW = 3.264 *p* = 0.353	*z* = 552.000 *p* = 0.945	*z* = 1943.500 *p* = 0.286	*z* = 3025.00 *p* = 0.098	***z*** = **3260.500** ***p*** = **0.035** *	*z* = 1649.000 *p* = 0.182	***z*** = **1613.500** ***p*** = **0.017** *
Self‐evaluation	***z*** = **2015.000** ***p*** = **0.005** *	***z*** = **2158.000** ***p*** = **0.008** *	KW = 3.509 *p* = 0.173	*z* = 1686.500 *p* = 0.823	*z* = 1072.000 *p* = 0.117	*z* = 648.500 *p* = 0.706	*z* = 2462.00 *p* = 0.763	*z* = 2094.000 *p* = 0.282	KW = 5.342 *p* = 0.128	*z* = 607.00 *p* = 0.637	***z*** = **2110.00** ***p*** = **0.027** *	*z* = 2860.500 *p* = 0.242	*z* = 3047.00 *p* = 0.135	*z* = 1897.000 *p* = 0.824	*z* = 2201.500 *p* = 0.831
Self‐reinforcing	***z*** = **2065.000** ***p*** = **0.031** *	***z*** = **2097.500** ***p*** = **0.013** *	KW = 1.853 *p* = 0.396	*z* = 1820.000 *p* = 0.645	*z* = 855.000 *p* = 0.878	*z* = 742.000 *p* = 0.689	*z* = 2769.500 *p* = 0.127	*z* = 2110.000 *p* = 0.323	KW = 0.506 *p* = 0.918	*z* = 717.00 *p* = 0.179	*z* = 2038.500 *p* = 0.127	***z*** = **3367.500** ***p*** = **0.003** *	***z*** = **3406.500** ***p*** = **0.008** *	*z* = 1842.00 *p* = 0.660	***z*** = **1691.000** ***p*** = **0.041** *

Abbreviations: KW, Kruskal–Wallis test; *t*, independent samples *t*‐test; *X* ^2^, chi‐square test; *z*, Mann–Whitney *U*‐test.

* *p* < 0.05; bold values, all bold values are true.

**TABLE 5:** Factor affecting infection prevention behaviours.

	Receiving influenza vaccine		Receiving pneumonia vaccine		Raw food		Delicatessen products		Eating salad in a café or restaurant		Consuming street food		Wearing a mask outside home	
	*B*	*p*	*B*	*p*	*B*	*p*	*B*	*p*	*B*	*p*	*B*	*p*	*B*	*p*
Age	0.076	0.118	0.967	**0.027** *	1.031	0.058	1.007	0.691	1.022	0.161	1.022	0.151	0.946	**0.002** *
Marital status	1.743	0.236	1.033	0.943	0.981	0.966	1.277	0.648	1.335	0.542	1.357	0.500	1.162	0.782
Self‐monitoring	1.623	0.137	1.161	0.632	1.995	**0.035** *	1.289	0.489	1.008	0.980	1.254	0.463	0.642	0.222
Self‐evaluation	0.504	**0.030** *	0.569	0.084	0.603	0.144	1.605	0.123	1.103	0.747	1.027	0.926	1.207	0.575
Self‐reinforcing	0.544	**0.036** *	0.562	**0.045** *	1.341	0.322	1.381	0.334	2.177	**0.011** *	1.767	**0.048** *	0.668	0.225
*R*	0.097		0.101		0.088		0.043		0.083		0.074		0.109	
*R* ^2^	0.132		0.134		0.123		0.068		0.113		0.098		0.158	
*p*	0.051		0.0404		0.054		0.019		0.003		0.005		0.006	

* *p* < 0.05.

## 4 Discussion

The present study showed that 50% of the kidney transplant recipients washed their hands, which is considered the leading infection prevention behaviour. According to research, 97% of patients pay attention to hand hygiene (Dolgun et al. 2017). However, the patients in the present study did not follow all the World Health Organization's recommendations about washing one's hands (WHO 2023) or the Infectious Diseases Guidelines of the American Society of Transplantation (Danziger‐Isakov and Kumar 2019), and, in particular, they were less likely to wash their hands every time they became dirty. This suggests that the patients lacked knowledge about hand hygiene, which underlines the need to enhance their knowledge and sensitivity regarding this issue.

It is recommended that organ transplant recipients have an influenza vaccine every year and a pneumonia vaccine every 5 or 10 years (Baker et al. 2017; Danziger‐Isakov and Kumar 2019; Voora and Adey 2019). In one study, 36% of the dialysis patients waiting for kidney transplantation were found to have had the pneumonia vaccine (Lee et al. 2016), while in another, 7% of the posttransplant recipients had had the vaccine (Chesi et al. 2009). Another study revealed that the rate of receiving a pneumonia vaccine among kidney transplant recipients was 2.5% (Bıyık 2019). It is expected that all transplant recipients have vaccines. However, in the present study, only 48% of the patients had had the influenza vaccine, while 60% of the patients had had the pneumonia vaccine.

With regard to the recommendations of the Infectious Diseases Community of Practice (IDCOP) of the American Society of Transplantation (Avery and Michaels 2019), it can be said that the patients in the present study partly adhered to nutrition hygiene. Nutrition hygiene‐related behaviours and risk factors vary greatly in different studies. A study by Lindup et al. (2020). found that among solid organ transplant recipients, 46% consumed cheese made from unpasteurised milk, 37% consumed raw delicatessen products, 31% consumed undercooked meat, 24% consumed products made from raw eggs, 15% consumed undercooked eggs and only 18% followed all the food safety recommendations. A study performed with lung transplant recipients demonstrated that 52% ate cheese from unpasteurised milk, while 93% avoided raw or undercooked eggs, 94% avoided products from unpasteurised milk and 100% routinely rinsed their fruit before eating (Jain et al. 2015). The nutrition hygiene‐related behaviours in organ transplant patients thus demonstrate many differences between different patients. These differences may have been caused by the difference between the variables studied, the patients' opportunities to access clean and safe water and food and a lack of training provided on this subject.

According to results from one study with kidney transplant recipients, 68% had their own room, 59% had their own toilet, 86% had their own bathroom, 87% had a separate room where they could stay when their families had guests, 38% always wore a mask at home and 75% wore a mask when they left home (Dolgun et al. 2017). Another study found that 9% avoided using mass transportation, 17% avoided contacting people with an infection and 6% did not go to crowded places (Hedayati, Shahgholian, and Ghadami 2017). In the present study, nearly all the recipients (92%) had information about sexually transmitted diseases. However, it has been emphasised in the literature that kidney transplant recipients usually consider counselling about sexuality offered by health professionals insufficient (Vranješ et al. 2022; Abarca‐Durán et al. 2021). In the present study, most of the patients (52%) preferred swimming in the sea, while less than half of the patients (45%) did not swim at all. The findings of the present study regarding the hygiene behaviours of the transplant recipients are both consistent with and conflict with various results in the literature. When the IDCOP guidelines are taken into consideration, the recipients in the present study can be considered to display partial adherence to personal hygiene behaviours to prevent infection (Avery and Micheals 2019). The difference between the variables studied in research may be related to the patients' lack of information when adapting to hygiene rules and their fear of infection.

In the present study, it was found that having a higher mean age had an effect on having the pneumonia vaccine and wearing a mask outside the home. Consistent with this finding, a study by Jain et al. (2015). showed that lung transplant recipients over the age of 40 years old paid more attention to safe health behaviours like hand hygiene and measures regarding the proper care of domestic animals. It can be suggested that as age increases, patients experience more comorbid diseases and are more attentive to health promotion behaviours. Unlike age, the time that had elapsed since transplantation was not shown to produce a difference in any infection prevention behaviours in the current study. In the literature, specific studies have argued that as the time after transplantation increases, adaptation to infection control recommendations both increases (Hedayati, Shahgholian, and Ghadami 2017) and decreases (Germani et al. 2011; Lindup et al. 2020). The inclusion of the patients a long time after transplantation (12.77 years) in the present study might have affected the lack of a difference in infection prevention behaviours. Additionally, gender did not cause a difference in infection prevention behaviours in the current study. While one other study showed no effect of gender on infection prevention behaviours (Jain et al. 2015), several studies found that women were more adherent to measures against infections (Dolgun et al. 2017; Germani et al. 2011; Hedayati, Shahgholian, and Ghadami 2017).

In the present study, it was found that the self‐management subscales were effective on not consuming raw food, having the influenza vaccine, having the pneumonia vaccine, not eating salad in cafés or restaurants and not consuming street food. Posttransplant patients have many self‐management‐related responsibilities, including controlling infection (hygiene behaviours and checking signs of infection), taking immunosuppressants, doing exercise, eating healthily and attending follow‐up visits regularly (Germani et al. 2011; Kuwaiti, Ghadami, and Yousefi 2017). Research has focused on explaining the dimensions of the concept of self‐management, but none of the studies so far have evaluated self‐management levels and their relation to adherence to protective health behaviours and, especially, to infection prevention behaviours. The findings of the present study support the hypothesis that a high level of self‐management in transplant recipients could produce a significant difference in their infection prevention behaviours.

In the present study, the infection prevention behaviours and self‐control/self‐management behaviours of the kidney transplant recipients were evaluated using face‐to‐face interviews and self‐report data collection tools. All the findings of the study might have been influenced by Hawthorne effect. The kidney transplant recipients may have consciously given answers that did not reflect their true behaviours. This could have produced a positive impact on the results. There is currently no valid and reliable tool to evaluate infection prevention behaviours of patients. Using a questionnaire developed by the researchers to examine infection prevention behaviours in this study can be considered as a limitation. Moreover, the study was performed with only kidney transplant recipients in a single centre. Therefore, the results of the study cannot be generalised to other organ transplant recipients. The study sample was heterogenous in terms of the time elapsing after kidney transplantation. Since data collection was performed at a single session with each patient, changes over time could not be shown prospectively. Finally, because the time since transplant was long, and since data about infection status were incomplete in the transplant centre where the study was conducted, posttransplant infection status could not be evaluated.

## 5 Conclusion

The present study revealed that the kidney transplant recipients displayed incomplete infection prevention behaviours, including hand hygiene. As their age increased, so did the rate of receiving the pneumonia vaccine. A higher rate of married patients reported rinsing vegetables compared to the single ones. Good self‐management was found to create a difference in infection prevention behaviours. It is clear that education programmes and interventions are needed to improve the infection prevention behaviours of kidney transplant recipients. The present study is the first to examine the difference in infection prevention behaviours of organ transplant patients in terms of self‐management and self‐control. However, it should be replicated in larger samples including with other organ transplant recipients in multiple organ transplantation centres.

## 6 Conflicts of Interest

The authors declare no conflicts of interest.

## Supporting information


Appendix S1.

## Data Availability

The authors have nothing to report.
